# Single-Cell RNA sequencing reveals immune cell dynamics and local intercellular communication in acute murine cardiac allograft rejection

**DOI:** 10.7150/thno.75543

**Published:** 2022-08-29

**Authors:** Zhang Chen, Heng Xu, Yuan Li, Xi Zhang, Jikai Cui, Yanqiang Zou, Jizhang Yu, Jie Wu, Jiahong Xia

**Affiliations:** 1 Department of Cardiovascular Surgery, Union Hospital, Tongji Medical College, Huazhong University of Science and Technology, Wuhan, China.; 2 Key Laboratory of Organ Transplantation, Ministry of Education; NHC Key Laboratory of Organ Transplantation; Key Laboratory of Organ Transplantation, Chinese Academy of Medical Sciences, Wuhan, China.

**Keywords:** Single-cell RNA transcriptomics, Acute rejection, Murine heart transplantation, Immune landscape, Intercellular communication

## Abstract

**Rationale:** Transplant rejection is a major impediment to long-term allograft survival, in which the actions of immune cells are of fundamental importance. However, the immune cell dynamics and local intercellular communication of acute cardiac allograft rejection are not completely clear.

**Methods:** Here we performed single-cell RNA sequencing on CD45^+^ immune cells isolated from cardiac grafts and spleens in a model of murine heterotopic heart transplantation. Moreover, we applied unsupervised clustering, functional enrichment analysis, cell trajectory construction and intercellular communication analysis to explore the immune cell dynamics and local intercellular communication of acute cardiac allograft rejection at single-cell level. The effect of CXCR3 antagonist and neutralizing antibody against its ligand on allograft rejection and T cell function was evaluated in murine heart transplantation model.

**Results:** We presented the immune cell landscape of acute murine cardiac allograft rejection at single-cell resolution, and uncovered the functional characteristics and differentiation trajectory of several alloreactive cell subpopulations, including Mki67^hi^ CTLs, Ccl5^hi^ CTLs, activated Tregs and alloreactive B cells. We demonstrated local intercellular communication and revealed the upregulation of CXCR3 and its ligands in cardiac allografts. Finally, CXCR3 blockade significantly suppressed acute cardiac allograft rejection and inhibited the alloreactive T cell function.

**Conclusions:** These results provide a new insight into the immune cell dynamics and local intercellular communication of acute cardiac allograft rejection, and suggest CXCR3 pathway may serve as a potential therapeutic target for transplant rejection.

## Introduction

Allograft rejection is a serious complication following heart transplantation that can eventually lead to cardiac graft dysfunction and failure 1. The course of allograft rejection is orchestrated by the cross-regulation of the adaptive and innate immune systems 2, 3. Various immune cells participate in transplant rejection, and the intercellular communication among immunocytes is also critical for the initiation and maintenance of allograft rejection. For instance, communication between T cells and antigen-presenting cells (APCs) is indispensable for the initiation of allograft rejection, and the interactions between chemokines and chemokine receptors are essential for the chemotaxis and differentiation of immunocytes. While traditional high-throughput sequencings (RNA and TCR sequencing) have offered substantial insight into transplant rejection in the past decade, some key questions cannot be addressed with these assays 4. First, traditional high-throughput sequencing does not address phenotypic heterogeneity, which is critical for recognizing cell subclusters. Second, these approaches do not provide a precise description of local intercellular communications, which is useful for understanding allograft rejection. Recently developed single‐cell transcriptomic technology characterizes gene expression across cell populations, which presents novel opportunities to overcome these limitations and study transplants in unprecedented detail 5.

Single-cell omic technologies, including single-cell RNA sequencing (scRNA-seq), single-cell TCR sequencing (scTCR-seq) and single-cell combined transcriptome and proteome sequencing (scCITE-seq), show great superiority in depicting immunological networks. These methods have provided much-needed information on immune dynamics in the tumour immune microenvironment and coronavirus disease 2019 (COVID-19) during disease progression and treatment. For example, the combination of multiple single-cell omic technologies identified various immunophenotypes and associated gene sets that were positively or negatively correlated with T cell expansion following anti-PD1 treatment in breast cancer 6. Immune responses show dramatic changes during the disease progression of COVID-19, both in the lungs and the peripheral blood, which were elucidated by single-cell omics 7, 8. In addition to its utility in cancer and COVID-19 research, single-cell omics also provides a new window into the immunological network of transplant rejection 4, 5. Several works have applied single-cell transcriptomic techniques in transplantation immunology, and some innovative findings have been reported. The immunological landscape of transplant arteriosclerosis was revealed by single-cell transcriptomics, and the CCL21/CXCR3 axis was identified as an important regulator of the immune response and might serves as a potential therapeutic target in disease treatment 9. However, the immune cell dynamics and local intercellular communications in acute murine cardiac allograft rejection are not yet fully understood, which require specific evaluation at single-cell level.

By analyzing single-cell transcriptomic datasets of immune cells isolated from murine heterotopic heart transplantation models, we gained insight into the dynamic immune activities in acute cardiac allograft rejection. First, we elucidated the immune cell landscape of acute murine cardiac allograft rejection at single-cell resolution, and eighteen distinct subclusters and six main cell types were identified. The immunological characteristics of several alloreactive subpopulations, including Mki67^hi^ cytotoxic T lymphocytes (Mki67^hi^ CTLs), Ccl5^hi^ cytotoxic T lymphocytes (Ccl5^hi^ CTLs), activated regulatory T cells (aTregs), and alloreactive B cells, were also uncovered. Second, we compared the local intercellular communication between two groups and found the upregulation of CXCR3 and its ligands in the allografts. Based on bioinformatic analysis, we further verified the high expression level of CXCR3 in allografts by flow cytometry, and CXCR3 blockade significantly suppressed acute cardiac allograft rejection. Furthermore, our single-cell transcriptomic data could become a useful resource for deeper and more comprehensive research on acute cardiac allograft rejection, which might allow the discovery of new therapeutic targets.

## Materials and methods

### Animals and murine heterotopic heart transplantation models

Eight to ten-week-old male C57BL/6J (B6; H-2b) and BALB/c (H-2d) mice were purchased from Charles River (Beijing, China). All mouse experiments were performed in a specific pathogen-free facility according to the guidelines of the animal care and use committee of Huazhong University of Science and Technology and complied with the National Institutes of Health (NIH) Guidelines for the care and use of laboratory animals. We established a murine heterotopic heart transplantation model as previously described 10, animals were anesthetized using 2% isoflurane and administered via nose cone mask. All animals were euthanized using CO_2_ asphyxiation, followed by cervical dislocation to obtain tissue samples. In the allograft group, BALB/c hearts were transplanted into fully MHC-mismatched B6 recipients. In the control group, B6 hearts were transplanted into MHC-matched B6 recipients.

### Cell isolation

Cardiac allografts normally arrest on Day 7 after transplantation because of acute rejection. It is reasonable to harvest cardiac grafts on Day 5, Day 6 or Day 7 before cardiac allografts arrest^11^, ^12^, which may depend on the experience of the research group. Therefore, we harvested spleens and cardiac allografts from allograft group recipients (n = 3) on Day 6 before cardiac allograft arrest. At the same timepoint, spleens and cardiac isografts were harvested from control group recipients (n = 3). CD45^+^ cells were respectively isolated after digestion, centrifugation and flow sorting. In brief, cardiac grafts were cut into pieces and digested with 1 mg/mL collagenase B (Roche 11088815001) in Hank's balanced salt mixture (Solarbio H1025, China) at 37 °C for 20 min, digestion solution was harvested and resuspended every five minutes. After digestion, the cell suspensions were collected and filtered through 70-μm cell strainers. Percoll (Solarbio P8370, China) was used to purify mononuclear cells through density centrifugation at 600 g and 25 °C for 25 min without braking. Splenocytes were prepared through grinding, erythrocyte lysis, washing and centrifugation (600 g, 3 min). Live CD45^+^ cells were then sorted by a BD FACSAria II flow cytometer after cardiac graft-infiltrating cells and splenocytes were stained with anti-CD45-APC (BioLegend 103112, USA) and a Zombie Green Fixable Viability Kit (BioLegend 423102, USA).

### scRNA-seq library preparation and sequencing

Single-cell gel bead-in-emulsions (GEMs) were constructed using a Chromium Single Cell 5' Library and Gel Bead Kit following the manufacturer's instructions. Briefly, FACS-sorted cells were washed with 0.04% BSA PBS three times and resuspended to a concentration of 700 ~ 1200 cells/µL (viability ≥ 85%), as determined by a Countess™ II Automated Cell Counter. The cells in the samples that reached the standard were then captured in droplets. Single-cell GEMs consisted of single-cell and specific 16-nt 10× barcodes and 10-nt unique molecular identifiers (UMIs). Within the single-cell GEMs, the tagged cells were lysed, and the released mRNA was barcoded. Reverse transcription (RT) was performed in the GEMs. After the RT step, the emulsion was broken, and amplification of the 10× barcoded cDNA was completed. Amplified cDNA was then used for the construction of 5′ gene expression libraries. Each cDNA library was sequenced on a NovaSeq platform (Illumina) to generate 150-bp paired-end reads.

### Preprocessing of scRNA-seq data

Raw feature counts were generated by the function 'CellRanger count' of Cell Ranger (10× Genomics, version 3.0.2). The input data were fastq files, and the mm10 genome was used as the reference genome. scRNA-seq expression matrices were generated by Seurat (version 4.0.1) 13, 14. Seurat offers functions for quality control, filtering, normalization and dimensionality reduction. To filter cells with low quality, we used the following selection criteria: cells with a gene number greater than 500 and less than 5000 and a mitochondrial DNA percentage lower than 5%, and genes with at least one feature count in more than 5 cells were selected for subsequent analysis.

### Cell clustering and cell type annotation

After preprocessing all scRNA-seq data, we integrated 4 groups using the 'FindIntegrationAnchors' and 'IntegrateData' functions. The abbreviations used to represent these groups were as follows: control group spleen (Ctrl-SP), allograft group spleen (Allo-SP), control group heart (Ctrl-HT), and allograft group cardiac graft (Allo-HT). Among them, control group heart was from GSE142564 15. We used the 'ScaleData' function to scale and centre the integrated data. After calculation with the 'RunPCA' function, the first 20 principal components were used for dimensionality reduction and clustering through 'FindClusters' functions (resolution = 0.6). The clusters were visualized with UMAP by seed.use = 3. The marker genes of each cluster were selected from differentially expressed genes (DEGs) using the 'FindAllMarkers' function. We annotated cell populations using a combination of automated software annotation and reference to recent high-throughput studies. The package SingleR (version 1.4.1) was used for cell type annotation based on the ImmGen database 16. Violin plots, feature plots, dot plots, and heatmaps of gene expression were generated with 'VlnPlot', 'FeaturePlot', 'DotPlot', and 'DoHeatmap', respectively.

### Marker gene identification and subsequent functional analysis

The DEGs of each group and marker genes of each cell cluster were identified by the Seurat function 'FindAllMarkers', with a minimum log-fold change threshold of 0.25, adjusted P values less than 0.05, and a minimum 0.25 fraction in cells. Functional annotation of DEGs was performed by the gene set enrichment analysis (GSEA) function in clusterProfiler version 3.18.1 17, with the threshold set to P-adjusted < 0.05. The gene sets used can be found in the Molecular Signatures Database (version 7.4) and Kyoto Encyclopedia of Genes and Genomes (KEGG). A volcano plot was generated in R with ggplot2 to show the genes with upregulated or downregulated expression.

### Construction of cell trajectories along the pseudo-time axis

After the cell data were passed through quality control, the R package monocle2 (version 2.18.0) was utilized to construct the pseudo-time trajectories of each cell type obtained from Seurat objects 18.

### Intercellular communication analysis

CellChat 19 is a new tool that is able to quantitively infer and analyse intercellular communication networks from scRNA-seq data. We used CellChat (version 1.0.0) to predict major signalling inputs and outputs for cells and how those cells and signals coordinated for functions using network analysis and pattern recognition approaches.

### Flow cytometry

Typically, surface staining was performed at room temperature for 15 min. Dead cells were excluded using a Zombie Aqua Fixable Viability Kit (BioLegend 423102, USA). For intracellular staining of cytokines, cells were restimulated with phorbol 12-myristate 13-acetate (PMA; 50 ng/ml, Abmole M4647, China) and ionomycin (500 ng/ml, Sigma-Aldrich 407951, USA), and cytokine secretion was blocked with GolgiStop (BD Biosciences 554724, USA) for 6 h according to the manufacturer's instructions. The cells were then fixed and permeabilized with a Fixation/Permeabilization Solution Kit (BD Biosciences 554715, USA), followed by staining with fluorochrome-labelled antibodies against cytokines according to the manufacturers' instructions. All samples were run on a BD LSRFortessa X-20 flow cytometer, and the results were analysed using FlowJo version 10 software. All antibodies and reagents used in our experiment are listed in Table S1.

### *In vivo* blockade of the CXCR3 pathway

For the *in vivo* CXCR3- and CXCL9-neutralizing experiments, heart transplant recipient mice received different treatments after surgery. In the anti-CXCL9 group, recipient mice were treated by intraperitoneal injection (i.p.) of 200 µg anti-CXCL9 monoclonal antibody (mAb) (BioXCell BE0309, clone: MIG-2F5.5, USA) every 2 days. In the anti-CXCR3 group, recipient mice were treated by i.p. injection of 200 µg anti-CXCR3 mAb (BioXCell BE0249, clone: CXCR3-173, USA) every 2 days. In the anti-CXCL9 + anti-CXCR3 group, recipient mice were treated by i.p. injection of 200 µg anti-CXCL9 mAb and 200 μg anti-CXCR3 mAb every 2 days until complete cessation of graft beating. In the isotype control group, recipient mice were treated by i.p. injection of 200 μg Armenian hamster IgG (BioXCell BP0091, USA).

### Statistical analysis

Data are expressed as the mean ± standard deviation (SD) and were analyzed by using GraphPad Prism 8 software. Two-tailed unpaired Student's t-test was used for comparisons between two groups. One-way ANOVA was used for comparisons among multiple groups. The P value for graft survival was determined by the Mann-Whitney test. The significance level was set at P < 0.05.

## Results

### scRNA-seq analysis reveals distinct cellular compositions in cardiac grafts and spleens

**Figure 1:**
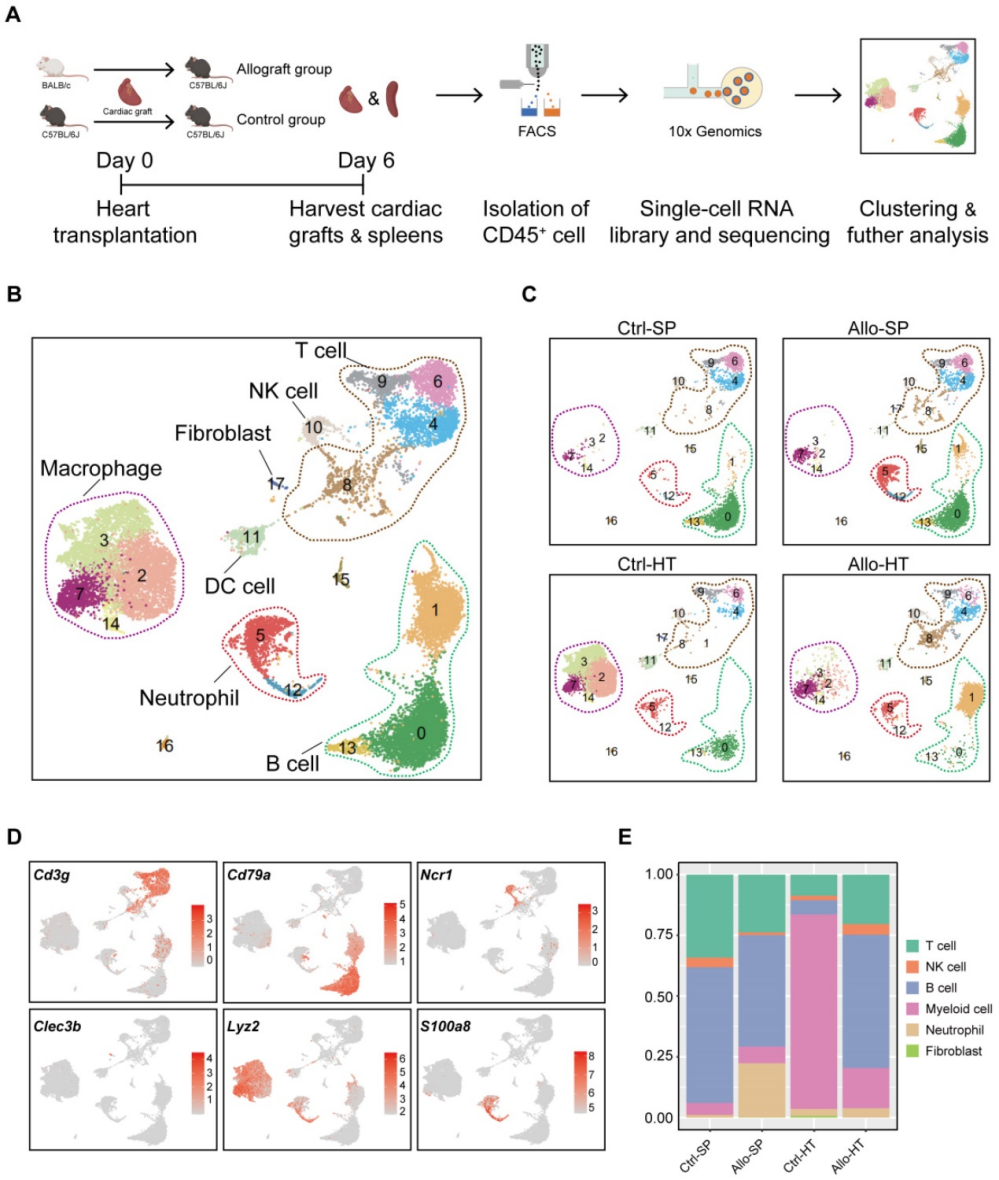
** Single-cell transcriptomics analysis reveals distinct cellular compositions in cardiac grafts and spleens. (A)** Schematic depicting heart transplantation model establishment and sample processing for single-cell RNA-seq. **(B)** Initial UMAP visualization of all cells (18,678) in 18 color-coded subclusters and 6 major cell types. **(C)** UMAP plots showing color-coded cell clusters in different groups. The abbreviations used to represent these groups were as follows: control group spleen (Ctrl-SP), allograft group spleen (Allo-SP), control group heart (Ctrl-HT), and allograft group cardiac graft (Allo-HT). **(D)** Feature plots of typical marker genes for 6 major cell types. **(E)** Bar chart demonstrating the proportions of major cell types among different groups.

As shown in Figure 1A, murine heterotopic heart transplantation models were established, including allograft group and control group. Among the mice, cardiac allografts (cardiac grafts in the allograft group) suffered acute rejection after transplantation because of MHC mismatching, but cardiac isografts (cardiac grafts in the control group) were free from rejection. This pattern was further confirmed by histological examination (Figure S1A-S1B). To characterize the immunocyte landscape, CD45^+^ cells were respectively isolated on Day 6 after transplantation. The gating strategy for flow sorting is depicted in Figure S1C. scRNA-seq analysis of the isolated CD45^+^ cells was then performed. After batch effect correction, the four datasets were well integrated (Figure S1D-S1G).

Unsupervised clustering results revealed various cell types in cardiac grafts and spleens, with a total of 18 cell subclusters and 6 main cell types identified based on typical marker genes and the top 5 variable genes (Figure 1B-1D, Figure S2A). As expected, we observed several distinct clusters in the allograft groups compared with the control groups (Figure 1C). The typical marker genes of 6 main cell types including *Cd3g* (T cells), *Cd79a* (B cells), *S100a8* (Neutrophils), *Ncr1* (NK cells), *Lyz2* (Myeloid cells), and *Clec3b* (Fibroblasts) are shown in Figure 1C. Significant changes and differences in cellular composition existed between the allograft group and control group for both the heart and spleen samples (Figure 1E, Figure S2B and S2C). Myeloid cell clusters accounted for over 80% of the total cells in the Ctrl-HT group; however, the proportion of myeloid cell clusters distinctly declined, ranging from 80.02% to 16.50%. Furthermore, the proportion of T cells increased from 8.74% to 20.37%, and the percentage of B cell clusters increased from 5.82% to 54.93% in the Allo-HT group. These great changes suggested central roles for T cell- and B cell-mediated adaptive immune responses in the complicated progression of acute heart transplant rejection. B cells and T cells represented over 90% of the total cells in the Ctrl-SP group. Although B cells and T cells remained the major cell types in the Allo-SP group, their percentages were decreased because of the infiltration of neutrophils. Distinct changes in the cellular compositions of cardiac grafts and spleens were uncovered by clustering analysis. Additional interesting phenomena may yet be revealed in further functional analyses of different cell types.

### T cells and NK cells mediate cellular immune responses in acute cardiac allograft rejection

**Figure 2:**
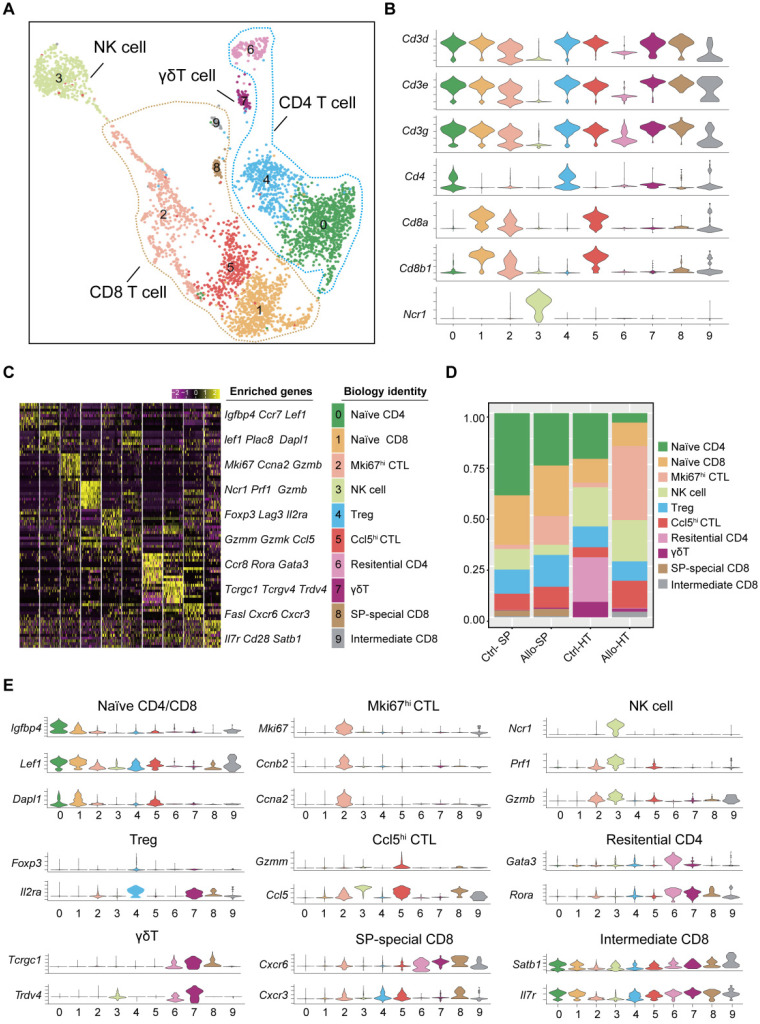
** Single-cell transcriptomics analysis present the dynamic of T and NK cells in acute murine cardiac allograft rejection. (A)** UMAP plots displaying ten color-coded subclusters of T and NK cells. **(B)** Violin plots showing the expression levels of typical marker genes (columns) defining T cell (*Cd3d*,* Cd3e*,* Cd3g*,* Cd4*, *Cd8a*, and *Cd8b1*) and NK cell (*Ncr1*) clusters (rows). **(C)** Heatmap depicting the expression of the top 10 enriched genes across cell clusters (columns), with typical enriched genes and putative biological identity. **(D)** Bar chart showing the proportion of each cell cluster among different groups. **(E)** Violin plots showing normalized expression levels of selected genes (columns) among ten clusters (rows).

Current studies commonly agree that the responses of T cells to alloantigen determine the short-term and long-term outcomes after solid organ transplantation 20, 21. It is valuable to explore the dynamic changes in T cell subsets and functionality in acute cardiac allograft rejection. Therefore, we reclustered 3605 T cells together with 496 NK cells at a higher resolution and identified nine T cell clusters (T cell subsets) and one NK cell population (Figure 2A). According to the transcriptomic characteristics of T cells and NK cells (Figure 2B), the ten cell subsets could be preliminarily divided into CD8^+^ T cells (Clusters 1, 2, 5, 8, and 9), CD4^+^ T cells (Clusters 0, 4, 6, and 7) and NK cells (Cluster 3). To correlate each cluster with a known T cell subset, we screened the top 10 variable genes of each cluster and compared them to those of previously reported T cell subsets and canonical markers 22 (Figure 2C and 2E).

**Figure 3:**
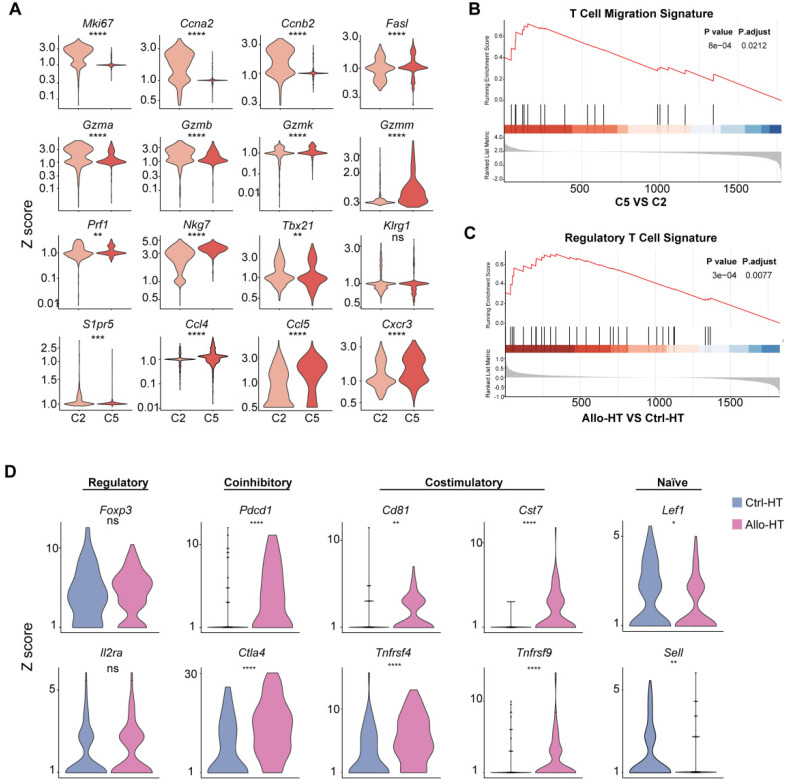
** Functional characteristics of alloreactive Mki67^hi^ CTLs, Ccl5^hi^ CTLs and Tregs in allografts. (A)** Violin plots showing normalized expression levels (Z scores) of selected genes in Cluster 2 and Cluster 5. **(B)** Gene set enrichment analysis (GSEA) comparing Ccl5^hi^ CTL (C5) to Mki67^hi^ CTL (C2) for T cell migration signature genes **(C)** GSEA comparing Treg cells in Allo-HT with Treg cells in Ctrl-HT for the regulatory T cell signature. **(D)** Violin plots showing the expression levels (Z scores) of selected genes among Treg cells in Ctrl-HT (blue) and Allo-HT (red). ns, not statistically significant. * *p* < 0.05, ** *p* < 0.01, *** *p* < 0.001, **** *p* < 0.0001.

For the four CD4^+^ T cell clusters, there was a population of naïve CD4^+^ T cells expressing high levels of naïve T cell-associated genes (*Lef1*,* Ccr7*, and* Igfbp4*) 22 (Cluster 0); a population of Tregs, labelled on the basis of their classic expression of the *Foxp3*,* Il2ra*, and* Lag3* (encoding the CD25 protein) genes (Cluster 4); a population of 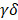
T cells overexpressing the* Tcrgc1*,* Tcrgv4*,* and Trdv4* genes (Cluster 7); and the Ctrl-HT-specific resident CD4^+^ T cell subpopulation, which showed high expression of the *Ccr8*,* Rora*, and* Gata3* genes (Cluster 6). Compared to that in the Ctrl-HT group, the Treg subpopulation in the Allo-HT group showed slight changes in absolute counts and relative proportions (Figure 2D, Figure S3B). However, the functional differences between the two groups were great. Compared to the Tregs in the Ctrl-HT group, the Tregs in the Allo-HT group overexpressed* Foxp3* and* Il2ra*, together with activated regulatory T cell (aTreg)-associated genes such as *Cd81* and *Cst7* 23, 24 and coinhibitory (*Pdcd1* and* Ctla4*) and costimulatory genes (*Tnfrsf4* and* Tnfrsf9*). The population of Tregs in the Ctrl-HT group was labelled on the basis of their classic expression of the* Foxp3* and* Il2ra* genes, together with the expression of the naïve-associated genes* Lef1* and *Sell* 22 (Figure 3D). The above results indicate that the Tregs in the Ctrl-HT group showed a resting Treg (rTreg) phenotype, while the Tregs in the Allo-HT group showed an aTreg phenotype. GSEA results also confirmed our conclusion (Figure 3C).

We next focused on CD8^+^ T cells, the most enriched T cell population in the Allo-HT (63.81%) and Allo-SP (53.19%) groups (Figure 2D, Figure S3B). Cluster 1 was defined as the naïve CD8^+^ T cell cluster, which expressed high levels of naïve T cell-associated genes (*Plac8*,* Dapl1*,* Lef1*, and* Igfbp4*) 22. Cluster 8 was defined as spleen-special CD8^+^ T cells. Cluster 9 appeared to be effector CD8^+^ T cells that highly express *Il7r*, *Statb1* and* Cd28* 25. Allograft group-enriched CD8^+^ T cells (Clusters 2 and 5) displayed high expression of granzyme genes (*Gzma*,* Gzmb*,* Gzmk*, and *Gzmm*) and expressed other cell cytotoxicity-related gene (*Prf1*), indicating that these subsets were CD8^+^ CTLs with potential cytotoxic ability 26. Cluster 2 also showed high expression of *Mki67*,* Ccna2*, and* Ccnb2* and was further annotated as Mki67^hi^ CTLs, while Cluster 5, which displayed high *Ccl5* expression, was annotated as Ccl5^hi^ CTLs 27. Further functional analysis and differential gene expression analysis suggested that showed stronger proliferative and cytotoxic killing abilities, whereas Ccl5^hi^ CTLs showed a stronger migratory capacity (Figure 3A and 3B).

### Features of alloreactive B cells in cardiac allografts

**Figure 4:**
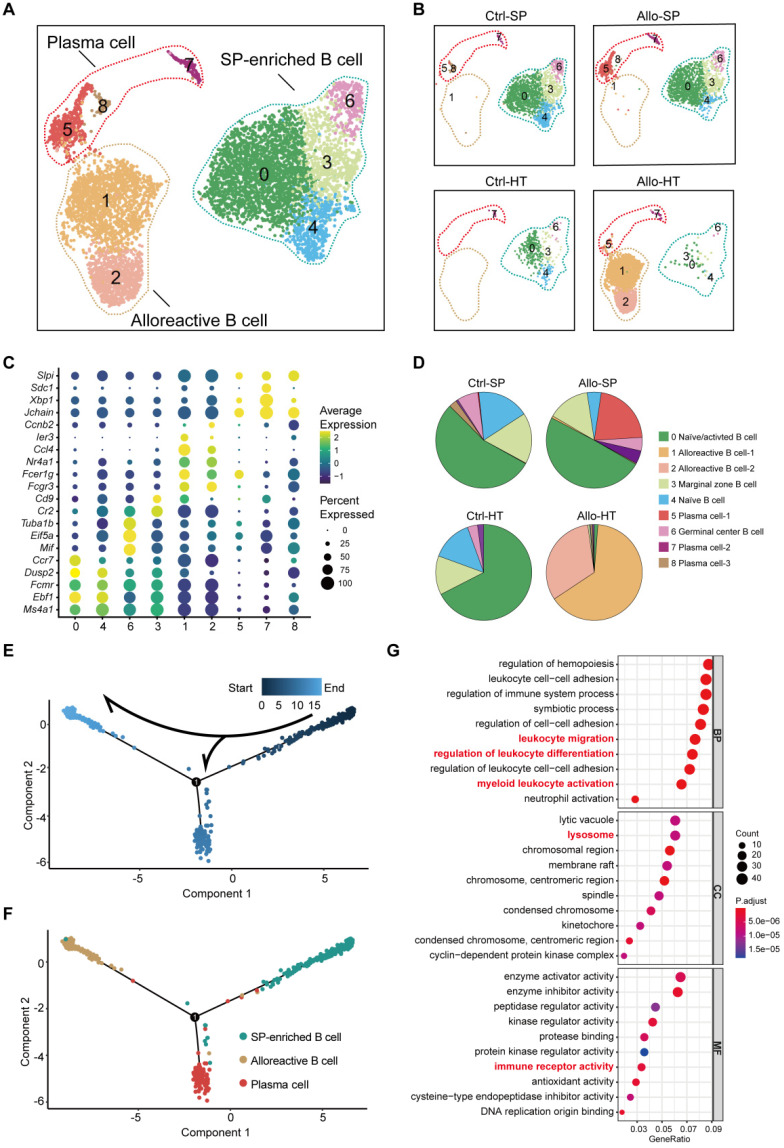
** Single-cell transcriptomics analysis present the dynamic of B cells in acute murine cardiac allograft rejection. (A)** UMAP plots displaying nine color-coded subclusters of B cells and three main cell types. **(B)** UMAP plots showing nine color-coded cell clusters in different groups. **(C)** Dot plot showing the expression levels (color-scaled, columnwise Z scores) of selected genes (columns)) and the percentage of expressing cells (dot size) among nine cell clusters (rows). **(D)** Pie charts demonstrating the proportion of nine subclusters in different groups. The colors of the pie charts are consistent with those in the UMAP plots. **(E)** Monocle pseudotime inference traces a path of three main cell types, with each color coded for pseudotime (top). **(F)** Differentiation trajectory of three main cell types, with each color coded for clusters (bottom). **(G)** Gene Ontology (GO) enrichment analysis of alloreactive B cells. BP: biological process; CC: cellular component; MF: molecular function.

B cells were initially considered to be antibody-producing cells in the pathological process of transplant rejection 28, but it has been recognized that B cells also act as APCs, capable of immune regulation and cytokine secretion 29, 30. To clearly describe the role of B cells in acute cardiac allograft rejection, high-resolution clustering analyses were performed. B cells were separated into nine subpopulations: two alloreactive B cell clusters (Clusters 1 and 2) were primarily enriched in the Allo-HT groups, and four clusters (Clusters 0, 3, 4, and 6) were identified as SP-enriched B cells that were mainly enriched in the Ctrl-SP and Allo-SP groups (Figure 4A and Figure 4B, Figure S4A). Differential gene expression analysis allowed us to define specific cell populations (Figure 4C, Figure S4B). Cluster 4 was characterized by marker genes of naïve B cells (*Ebf1*,* Dusp2*,* Fcmr*, and *Ms4a1*). Cluster 0 comprised a mix of naïve and activated cells expressing *Ms4a1*,* Ebf1*,* Fcmr*,* Ccr7*, and* Dusp2* 31, 32. It is possible that these cells were also partially activated *in vivo*. Marginal zone B cells (Cluster 3) showed high expression of the marker genes *Cd9* and* Cr2* 32. In Cluster 6, marker genes of germinal centre B cells (*Eif5a*,* Mif*, and *Tuba1b*) were highly expressed 33, 34. We identified two Allo-HT group-enriched clusters (Clusters 1 and 2) that expressed genes associated with activated B cells (*Fcgr3*,* Fcer1*,* Nr4a1*,* Ccl4*,* Ier3*, and *Ccnb2*) 32, 35. Plasma cell clusters (Clusters 5, 7 and 8) were identified by canonical markers (*Jchain*,* Sdc1*,* Xbp1*, and* Slpi*) 36.

The B cell compositions in the Allo-SP and Ctrl-SP groups were similar, and spleen-enriched clusters were the most abundant B cell subsets in these two groups (Figure 4B and 4D). However, plasma cells (Clusters 5 and 7) were significantly increased in the Allo-SP group, which indicated a possible continuous humoral immune response in acute cardiac allograft rejection. Two clusters (Clusters 1 and 2) existed almost exclusively in the Allo-HT group and could be considered alloreactive B cells in cardiac allografts. To better understand the transcriptional dynamics of different B cell populations, we constructed the cell trajectory of each cell type over pseudo-time (Figure 4E and 4F, Figure S4C). Significantly, we found that the SP-enriched clusters (Clusters 0, 3, 4 and 6) were placed early in the trajectory, whereas alloreactive B cell clusters (Clusters 1 and 2) and plasma cell clusters (Clusters 5, 7, and 8) were placed at the two terminals of the trajectory. This confirmed that the majority of the B cells in the Allo-SP group were still quiescent in an acute cardiac allograft rejection environment but that alloreactive B cells infiltrating cardiac allografts were activated. The pathway enrichment results also demonstrated the activity state of alloreactive B cells. Alloreactive B cells showed a stronger migratory ability and might play an important role in leukocyte differentiation regulation (Figure 4G).

### High-resolution analyses reveal distinct cell populations involved in the innate immune response

**Figure 5:**
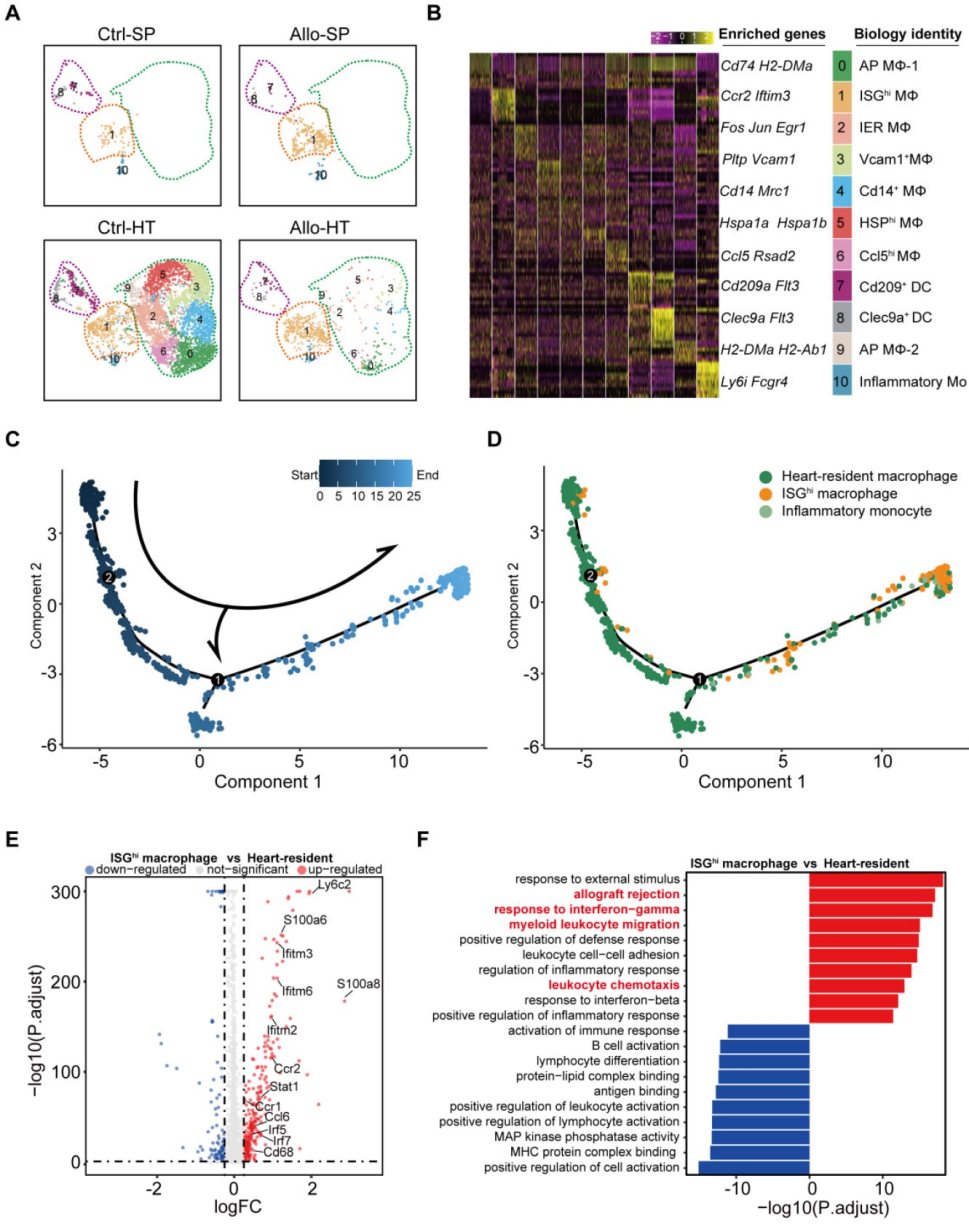
** Focused analyses of ISG^hi^ macrophages enriched in allografts. (A)** UMAP plots showing eleven color-coded cell subclusters in different groups. **(B)** Heatmap demonstrating the expression of the top 10 enriched genes across cell clusters (columns), with typical enriched genes and putative biological identity. **(C)** Monocle pseudotime inference traces a path along three main cell types, with each color-coded by pseudotime (top). **(D)** Differentiation trajectory of three main cell types, with each color-coded by cluster (bottom). **(E)** Volcano plots showing gene features between ISG^hi^ macrophages and heart-resident macrophages. DEGs (with -log10P > 4; logFC (fold change) > 0.5 or logFC (fold change) < -0.5) are highlighted in red or blue. **(F)** Differences in the top 10 enrichment pathways between ISG^hi^ macrophages (red) and heart-resident macrophages (blue).

Decades of research have established that the adaptive immune system is critical in acute transplant rejection, but accumulating evidence indicates that the innate immune system also plays an important role in acute transplant rejection 2. Our previous results suggested that myeloid cells (including monocytes, macrophages, and dendritic cells (DCs) accounted for the majority of immune cells in the Ctrl-HT group, and the proportion of myeloid cells in the Allo-HT group was significantly decreased (Figure 1E). To obtain more information on the dynamic changes in myeloid cells, we performed focused clustering analysis of these cells at a higher resolution and separated them into eleven clusters (Figure S5A, Figure 5A). The difference between the Ctrl-HT and Allo-HT groups was large, and the quantity of myeloid cells decreased dramatically in the Allo-HT group, with the exception of Cluster 1 and Cluster 10 (Figure 5A). Differential gene expression analysis allowed us to define cell populations (Figure 5B, Figure S5C). Cluster 1 was most similar to an interferon-responsive (ISG^hi^) population because it showed strong expression of interferon-responsive signature-associated genes (*Ifitm2*,* Ifitm3*,* Ifitm6*, and* Irf7*) and the proinflammatory genes *S100a6* and *S100a11*. Seven clusters (Clusters 0, 2, 3, 4, 5, 6, and 9) were identified as heart-resident macrophages that were mainly observed in the Ctrl-HT group. Cluster 0 was identified as antigen-presenting macrophages (AP MΦ-1) and was characterized by high expression of antigen presentation-associated genes (*H2-DMb2*,* Cd83*,* H2-Ab1*,* H2-Eb1*,* H2-Aa*,* Cd74*, and *Cd40*). Cluster 9 was also defined as antigen-presenting macrophages (AP MΦ-2) with high expression of *H2-DMa*, *H2-Ab1*,* H2-Eb1*,* H2-Aa*, and* Cd74.* Cluster 2 was characterized by high expression of immediate early genes (Jun, Fos, Egr1, Klf2, and Aft3) encoding transcription factors 37 and annotated as immediate early response macrophages (IER MΦs). Cluster 3 was suggested to be Vcam1^+^ macrophages (Vcam1^+^ MΦ) 38, with high expression of Vcam1. Cluster 4 was defined as Cd14^+^ macrophages without other highly expressed genes. Cluster 5 was characterized by high expression of heat shock protein (HSP) genes (*Hspa1a*,* Hspa1b*, and *Hsph1*) and annotated as HSP^hi^ MΦ 39. Cluster 6 was defined as Ccl5-high macrophages (Ccl5^hi^ MΦs) with high expression of *Ccl5* and *Rsad2*. Two clusters (Clusters 7 and 8) were defined as DC clusters that overexpressed *Cst3* and* Flt3.* Cluster 7 was suggested to be Cd209^+^ DCs, with high expression of Cd*209* 40. Cluster 8 was annotated as Clec9a^hi^ DCs, which exhibited high expression of *Clec9a* 41. Cluster 10 was most similar to inflammatory monocytes, with expression of *Ly6i* and the monocyte markers *CD14* and *Fcgr3.*

Cell trajectory analysis demonstrated that ISG^hi^ macrophages (Cluster 1, enriched in the Allo-HT group) represented the terminal stage of the trajectory, and most heart-resident macrophages were placed early in the trajectory (Figure 5C and 5D). This result traced a putative differentiation path from heart-resident macrophages to ISG^hi^ macrophages. Further analysis of the DEGs between ISG^hi^ macrophages and heart-resident macrophages also confirmed the proinflammatory and interferon-responsive phenotype of ISG^hi^ macrophages (Figure 5E). In addition, macrophage migration-associated genes (*Ccl6*,* Ccr2*, and *Ccr1*) were upregulated in ISG^hi^ macrophages. The top 10 enriched Gene Ontology biological process terms (GOBPs) in ISG^hi^ macrophages further supported the identity of this cluster, as these genes were involved in allograft rejection, myeloid leukocyte migration, leukocyte chemotaxis, response to interferon-gamma, and response to interferon-beta (Figure 5F).

### Local intercellular communication analysis identifies the CXCR3 pathway as a potential therapeutic target

**Figure 6:**
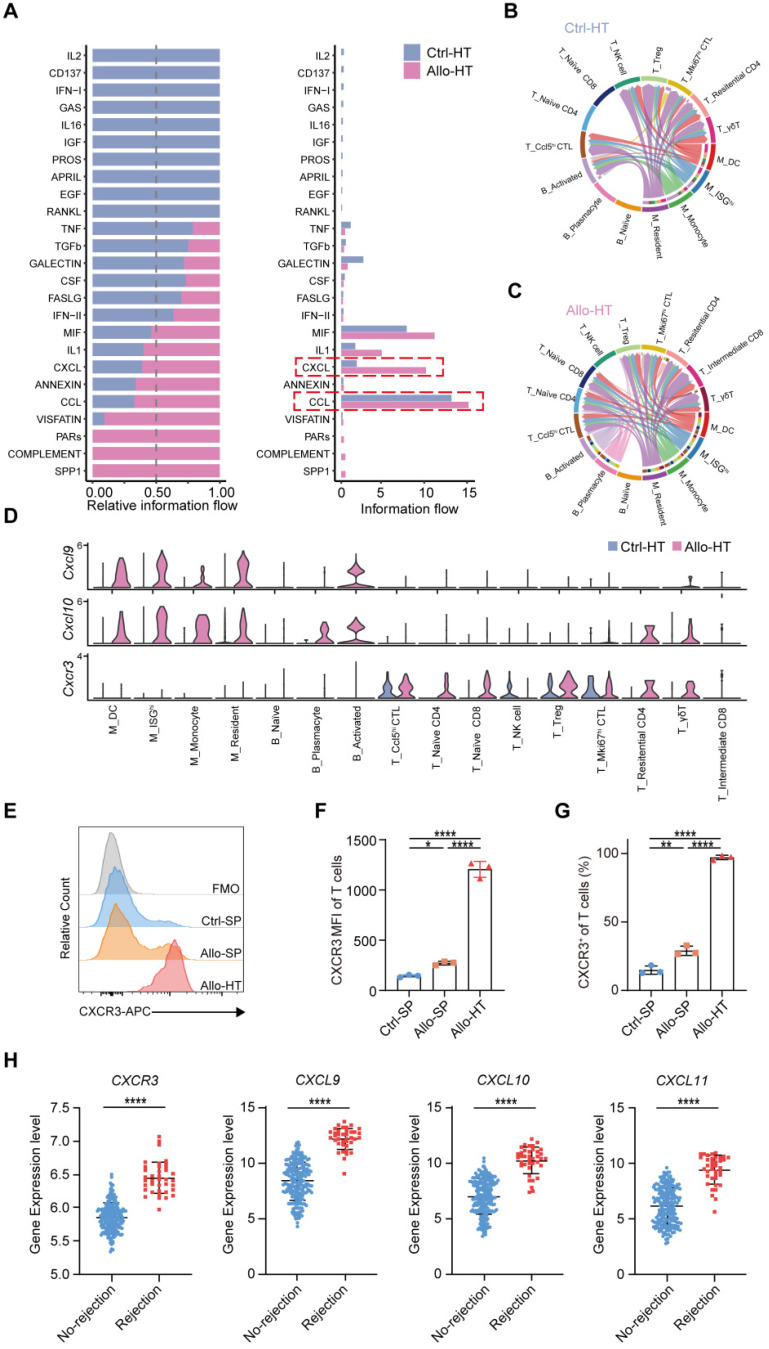
** Local intercellular communication analysis discloses the activation of CXCR3 pathway in acute murine cardiac allograft rejection. (A)** All significant signaling pathways were ranked based on their differences in overall information flow within the inferred networks between Ctrl-HT and Allo-HT. The top signaling pathways (colored blue) are more enriched in Ctrl-HT, and the bottom signaling pathways (colored red) are more enriched in Allo-HT. **(B)** CXCL signaling pathway network in Ctrl-HT. **(C)** CXCL signaling pathway network in Allo-HT. **(D)** Expression distribution of CXCR3 signaling genes (*Cxcr3*,* Cxcl9*, and* Cxcl10*) at Ctrl-HT (blue) and Allo-HT (red). **(E)** The expression level of CXCR3 in T cells was assessed by Flow cytometry **(F)** Bar plots show the mean fluorescence intensity (MFI) of CXCR3 in T cells (n = 3). **(G)** Bar plots show the positive proportion of CXCR3^+^ T cells in different groups (n = 3). **(H)** Bar plots show the relative expression level of *CXCR3*, *CXCL9*,* CXCL10*,* and CXCL11* in human heart transplant endomyocardial biopsies (n = 210 in No-rejection group, n = 38 in rejection group). All data are representative of three independent experiments at least. One-way ANOVA was used for comparisons among multiple groups. Two-tailed unpaired Student's t-test was used for comparisons between two groups. Data represented as the mean ± SD. * *p* < 0.05, ** *p* < 0.01, **** *p* < 0.0001.

Compared to bulk RNA-seq, scRNA-seq has the advantage of allowing the exploration of cellular signalling at the individual cell level and consequently the identification of potentially novel intercellular communications. To explore local intercellular communication changes during acute cardiac transplant rejection, we compared cellular signalling between the Allo-HT and Ctrl-HT groups (Figure 6A). We found that CXCL - CCL signalling pathways were more abundant in the Allo-HT group (Figure 6B and 6C, Figure S6A and S6B). Further analysis of CXCL-CCL signalling pathway networks revealed that *Cxcr3* and its ligands *Cxcl9* and *Cxcl10* were significantly highly expressed in the Allo-HT group (Figure 6D). *Cxcl9* and *Cxcl10* were mainly enriched in myeloid cell clusters and B cell clusters in the Allo-HT group, but *Cxcr3* was mainly enriched in T cell clusters in this group. The high expression level of CXCR3 in allograft-infiltrating T cells was also confirmed by flow cytometry (Figure 6E-6G). We also assessed the expression level of *CXCR3*,* CXCL9*,* CXCL10*,* and CXCL11* in clinical database which including 331 human heart transplant endomyocardial biopsies 42, and these genes were also upregulated in rejection group (Figure 6H). These results indicate that the CXCR3 pathway is significantly involved in acute heart transplant rejection and might be a potential therapeutic target.

### Blocking the CXCR3 pathway prolongs allograft survival and inhibits T cell activation in a murine heart transplantation model

**Figure 7:**
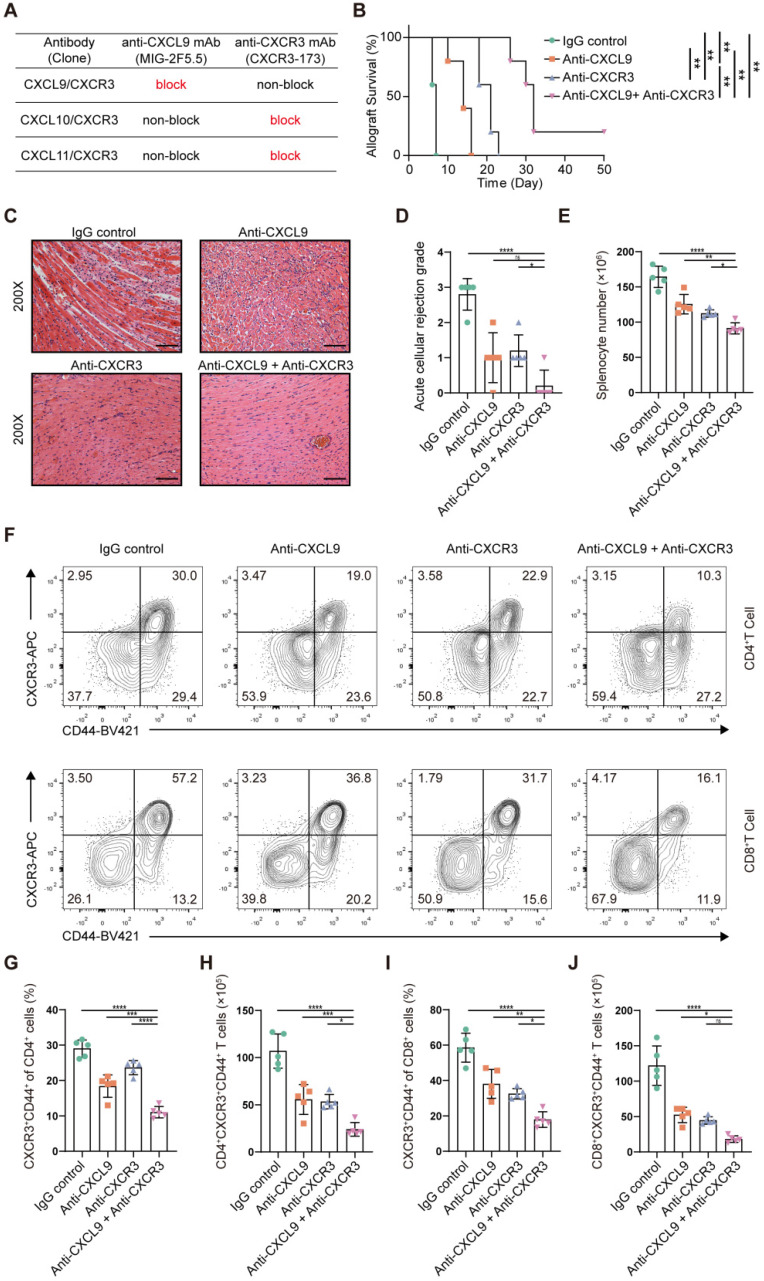
** Blockade of the CXCR3 pathway prolongs allograft survival and reduces CD44^+^CXCR3^+^ T cells in the murine heart transplantation model. (A)** Table shows the axis blocked by CXCR3-173 mAb or MIG-2F5.5 mAb. **(B)** Kaplan-Meier curves of cardiac allograft survival in different groups (n = 5). **(C)** Representative H&E-stained sections of cardiac allografts at day 6 post-transplant (n = 5, 200×, Scale bars: 100 µm). **(D)** Bar plots show the acute cellular rejection grade of cardiac allografts in different groups at day 6 post-transplant. **(E)** The total number of splenocytes in different groups at day 6 post-transplant (n = 5). **(F)** Representative FCM plots showing the percentage of activated T cells (CD44^+^ CXCR3^+^) in CD4 and CD8 T cells. Samples are splenocytes harvested at day 6 post-transplant. **(G)** Bar plots show the percentage of CD44^+^ CXCR3^+^ T cells in CD4 T cells (n = 5). **(H)** Bar plots show the number of CD4^+^ CXCR3^+^ CD44^+^ T cells (n = 5). **(I)** Bar plots show the percentage of CD44^+^ CXCR3^+^ T cells in CD8 T cells (n = 5). **(J)** Bar plots show the number of CD8^+^ CXCR3^+^ CD44^+^ T cells (n = 5). All data are representative of three independent experiments at least. One-way ANOVA was used for comparisons among multiple groups. The Mann-Whitney test was used for Kaplan-Meier curve comparisons. Data represented as the mean ± SD. ns, not statistically significant. * *p* < 0.05, ** *p* < 0.01, *** *p* < 0.001, **** *p* < 0.0001.

CXCR3, the receptor for the chemokines CXCL9, CXCL10 and CXCL11, is highly expressed on activated T cells. However, there is no commercially available antibody that can completely block it. The CXCR3-173 mAb is a monoclonal antibody that recognizes an epitope of CXCR3 and significantly inhibits binding to CXCL10 (IP-10) and CXCL11 (ITAC) but not to CXCL9 (MIG) 43. Thus, the CXCR3 pathway could not be completely blocked by the CXCR3-173 mAb. To verify the effect of blocking the CXCR3 pathway in acute murine cardiac allograft rejection, we combined the CXCR3-173 mAb with the MIG-2F5.5 mAb to completely block the CXCR3 pathway (Figure 7A). After murine allograft heart transplantation models were established, recipients were randomly divided into four groups that received different treatments. The four groups included one isotype control group (IgG control group) and three experimental groups (anti-CXCL9 group, anti-CXCR3 group, and anti-CXCL9 + anti-CXCR3 group). Compared with those in the control group, the recipients in the three experimental groups had longer allograft survival (Figure 7B). The median survival times of the anti-CXCL9 group, anti-CXCR3 group and anti-CXCL9 + anti-CXCR3 group were 14 days, 21 days and 32 days, respectively (Figure 7C). These results indicated that targeting the CXCR3 pathway could protect against acute heart transplant rejection and that blocking the CXCR3 pathway completely achieved better results. Histological evaluation of cardiac allografts harvested from recipients also showed that the anti-CXCL9 + anti-CXCR3 group had a minimal acute cellular rejection grade (Figure 7D and 7E). Next, we analyzed splenocytes from recipients in the four groups by using flow cytometry (Figure S7A). Before further analyses, we determined the relative proportions and absolute counts of CD4^+^ and CD8^+^ T cells, which could affect related statistical indicators (Figure S7B-S7F). As shown in Figure 7F, effector (CD44^+^CXCR3^+^) CD4^+^ T cells and effector (CD44^+^CXCR3^+^) CD8^+^ T cells were notably reduced in the three experimental groups in comparison with the control group, in terms of both relative proportions (Figure 7G and 7I) and absolute numbers (Figure 7H and 7J), and the anti-CXCL9 + anti-CXCR3 group showed the most remarkable reductions among the three experimental groups. These results indicated that targeting the CXCR3 pathway significantly suppressed T cell activation.

In addition, we detected the effector function of T cells, including IFN-γ^+^CD4^+^ T cells and IFN-γ^+^CD8^+^ T cells, in different groups (Figure S8A). Compared with the IgG control group, the anti-CXCL9 + anti-CXCR3 group exhibited significant reductions in IFN-γ^+^CD4^+^ T cells (Figure S8B and S8E) and IFN-γ^+^CD8^+^ T cells (Figure S8D and S8G). We next assessed the impact of targeting the CXCR3 pathway on Tregs (CD4^+^FOXP3^+^), and the results were negative (Figure S8C and S8F). Taking all the above results together, we propose that completely blocking the CXCR3 pathway significantly prolongs allograft survival and inhibits T cell activation.

## Discussion

In this study, we performed scRNA-seq to characterize the heterogeneous immune cell populations in cardiac grafts and spleens using a murine heterotopic heart transplantation model. Single-cell transcriptomic analysis identified various subpopulations of T cells, B cells and myeloid cells. We described dynamic changes in the proportions of cell subsets and further analyzed features of specific alloreactive subpopulations. The details of immune cell dynamics and intercellular communications provided by scRNA-seq could be helpful for further understanding transplant rejection and exploring therapeutic targets.

Single-cell omic techniques provide new approaches to explore critical and challenging questions in transplantation: the definition of crucial cell clusters involved in protective or pathogenic responses, the identification of donor- and recipient-derived immune cell infiltrates in the allograft, and the detection of the mechanisms underlying allograft rejection 4. Several researchers have applied single-cell omics in transplant research for different scientific purposes, such as characterizing the cell atlas of transplant rejection 15, 44-46, describing the features of graft-infiltrating cells^47^, detecting potential targets in transplant rejection^48^, ^49^, and revealing the dynamic immune features of transplant recipients receiving immunosuppressive treatment 50. Our research filled this gap by utilizing single-cell transcriptomics to explore graft-infiltrating cell dynamics and local intercellular communication during acute murine cardiac allograft rejection. We annotated the transcriptomic features of T, B and myeloid cell clusters during acute heart transplant rejection in detail. Further functional and cell trajectory analyses also revealed the differentiation sources and functional features of alloreactive cell clusters. Among the T cell clusters, there were two alloreactive CD8^+^ clusters (Mki67^hi^ CTLs and Ccl5^hi^ CTLs) that were markedly increased in the allograft groups. These two clusters both possessed a cytotoxic ability but with different features. Mki67^hi^ CTLs showed stronger proliferative and cytotoxic killing abilities, while Ccl5^hi^ CTLs showed a stronger migratory capacity. Among the B cell clusters, two clusters (activated B cells 1 and 2) existed almost exclusively in cardiac allografts and could be considered alloreactive B cells. Alloreactive B cells showed a stronger migratory ability and might play an important role in leukocyte differentiation regulation. In the myeloid cell clusters, the macrophage cluster enriched in allografts was an interferon-responsive population that showed a proinflammatory and migratory phenotype.

In addition to describing immune cell population dynamics and gene expression changes, single-cell transcriptomics can support the exploration of local intercellular communication at the individual cell level. The pathway changes in local intercellular communication provide a reference for designing therapies to suppress allograft rejection. We compared the local intercellular communication between allografts and isografts and found the upregulation of the CXCR3 pathway in the murine cardiac allografts. The same upward trend also exited in human heart transplant endomyocardial biopsy specimens which suffered acute transplant rejection. CXCR3, the receptor for the chemokines CXCL9, CXCL10 and CXCL11, is highly expressed on activated T cells. The CXCR3 signaling pathway plays essential roles in the cellular differentiation and migratory function of T cells 51. The role of the CXCR3 signaling pathway in allograft transplant rejection has been explored in several previous studies 43, 52-56. In several clinical researches, CXCR3 and its ligands were considered as potential diagnostic markers of allograft rejection. For example, Dany Anglicheau investigated that the urinary chemokines CXCL9 and CXCL10 are promising noninvasive diagnostic markers of acute rejection in kidney recipients. John A. Belperio and colleagues evaluated the correlation between bronchoalveolar lavage fluid CXCR3 chemokines with episodes of acute rejection, acute lung injury in lung transplant recipients. In addition, the majority of basic studies suggest that blocking the CXCR3 pathway suppressed acute cardiac allograft rejection, but there is one exception. Zerwes et al. reported that *Cxcr3* deficiency in recipients did not diminish graft infiltration or rejection, suggesting that passenger leukocytes expressing CXCR3 might be involved in rejection or that other effector molecules support compensatory proliferation to compensate for the deficiency in CXCR3. Nevertheless, all previous research targeted only one molecule (receptor or ligand) in the CXCR3 signaling pathway, which is insufficient to fully block the CXCR3 pathway. To compensate for this experimental defect, we combined the CXCR3-173 mAb and MIG-2F5.5 mAb to completely block this pathway in our murine heart transplantation model. The results demonstrated that the combination of the CXCR3-173 mAb and MIG-2F5.5 mAb could limit acute rejection significantly more than a single neutralizing antibody. This provides a new approach for rejection treatment and drug development, and a fully blocking antibody targeting the CXCR3 pathway is urgently needed.

However, our study had some limitations. We harvested grafts and spleens only on day 6, so our observations cannot be generalized across the whole dynamic process (from day 0 to cardiac allograft arrest) of acute heart transplant rejection. Clearly, further studies are required to describe the dynamic immune profiles of acute heart transplant rejection.

In conclusion, this study provides a new perspective for understanding acute cardiac transplant rejection at single-cell resolution. Our single-cell transcriptomic data could become a useful resource for deeper and more comprehensive research on acute heart transplant rejection, which might enable the discovery of new therapeutic targets.

## Supplementary Material

Supplementary figures and table.
