# Supplementary material for: Single-Cell RNA sequencing reveals immune cell dynamics and local intercellular communication in acute murine cardiac allograft rejection
Source: Theranostics. 2022 Aug 29;12(14):6242–57. doi: 10.7150/thno.75543 (PMC9475451; doi:10.7150/thno.75543)
Supplement: Supplementary file 1 — Supplementary figures and table. [file thnov12p6242s1.pdf]

Single-Cell RNA sequencing reveals immune cell dynamics and local intercellular communication in acute murine cardiac allograft rejection

Zhang Chen[1,2#], Heng Xu[1#], Yuan Li[1,2#], Xi Zhang[1], Jikai Cui[1,2], Yanqiang Zou[1], Jizhang Yu[1,2*], Jie Wu[1,2*], Jiahong Xia[1,2*]

[1]Department of Cardiovascular Surgery, Union Hospital, Tongji Medical College, Huazhong University of Science and Technology, Wuhan, China

[2]Key Laboratory of Organ Transplantation, Ministry of Education; NHC Key Laboratory of Organ Transplantation; Key Laboratory of Organ Transplantation, Chinese Academy of Medical Sciences, Wuhan, China.

#Zhang Chen, Heng Xu and Yuan Li contributed equally to this work.

*Correspondence: Jiahong Xia, E-mail: jiahong.xia@mail.hust.edu.cn or Jie Wu, E-mail: wujie426@hust.edu.cn or Jizhang Yu, E-mail: 2020508076@hust.edu.cn

Contents

Figure S1 2
Figure S2 4
Figure S3 5
Figure S4 6
Figure S5 7
Figure S6 8
Figure S7 9
Figure S8 10
Table S1 12

Figure S1

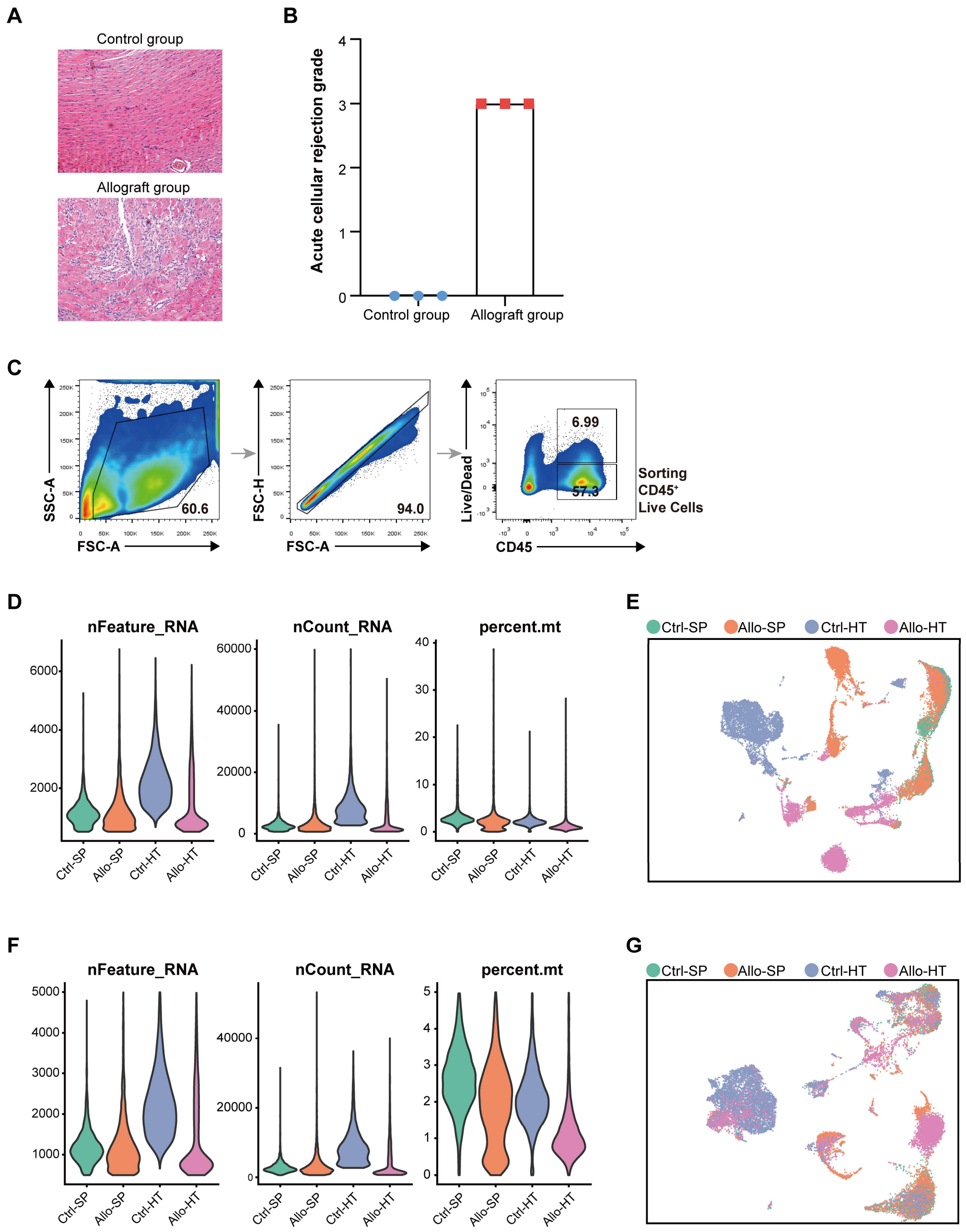

Figure S1. (Related to Figure 1) Sample preparation and data integration.

(A) H&E staining confirmed the occurrence of acute transplant rejection. (B) Bar plots show the acute cellular rejection grade of cardiac allografts in isograft group and allograft group (n = 3). (C) Gating strategy of CD45^{+}live cells sorting for single-cell RNA sequencing library. (D) Before batch effect corrected, scatterplot illustrating the number of genes, unique molecular identifiers (UMIs) and percentage of mitochondrial

genes in the four groups. (E) UMAP visualization of all cells before batch effect corrected. (F) After batch effect corrected, scatterplot illustrating the number of genes, unique molecular identifiers (UMIs) and percentage of mitochondrial genes in the four groups. (G) UMAP visualization of all cells after batch effect corrected.

Figure S2

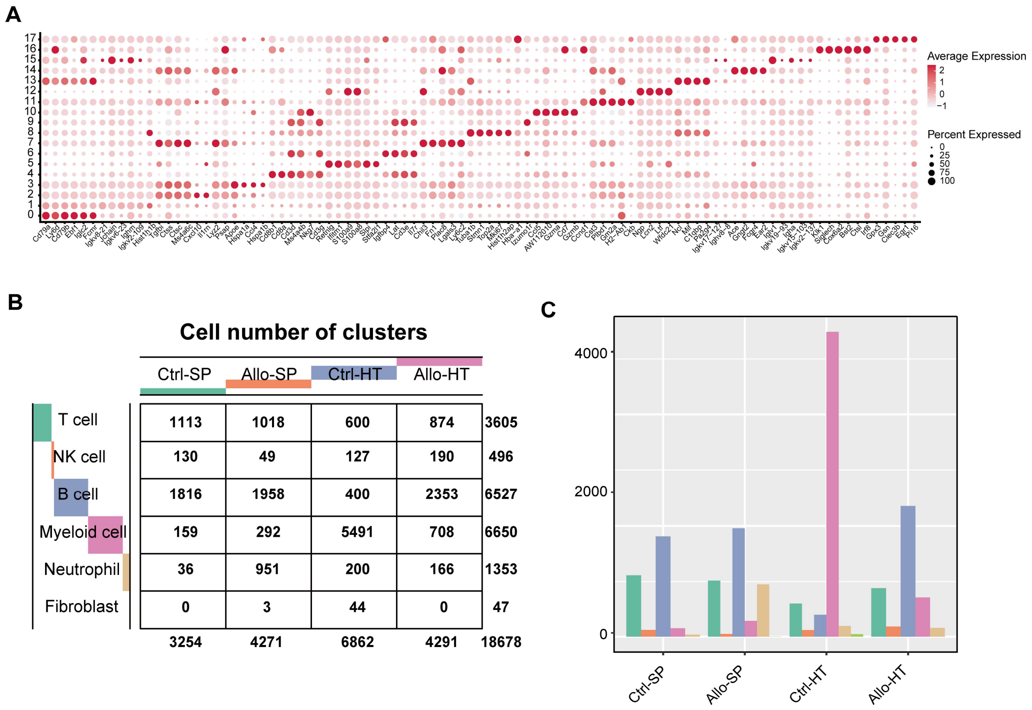

Figure S2. (Related to Figure 1) Top 5 DEGs across identified cell clusters and cell number of five main cell types.

(A) Dot plot displaying average scaled expression levels (color-scaled, column-wise Z scores) of top 5 DEGs (rows) across identified cell clusters (columns). Dot size reflects the percentage of cells expressing the selected gene in each cell cluster. (B) Absolute counting of six main cell types(columns) in four groups (rows). (C) Histogram of six main cell types absolute counting in four groups, colors of histograms are consistent with Figure S2B.

Figure S3

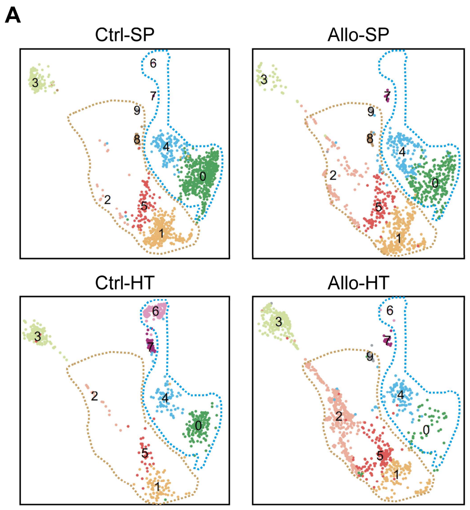

B

Cell number of clusters

	Ctrl-SP	Allo-SP	Ctrl-HT	Allo-HT	
0	490	257	151	49	947
1	295	249	79	120	743
2	26	141	15	381	563
3	122	50	130	212	514
4	144	157	69	100	470
5	97	103	33	140	373
6	1	0	148	3	152
7	2	6	51	16	75
8	34	35	0	0	69
9	7	6	0	29	42
	1218	1004	676	1050	3948

Figure S3. (Related to Figure 2) Focused analysis of T and NK cells

(A) UMAP plots showing ten color-coded subclusters of T and NK cells in different groups. (B) Absolute counting of ten color-coded subclusters (columns) in four groups (rows).

Figure S4

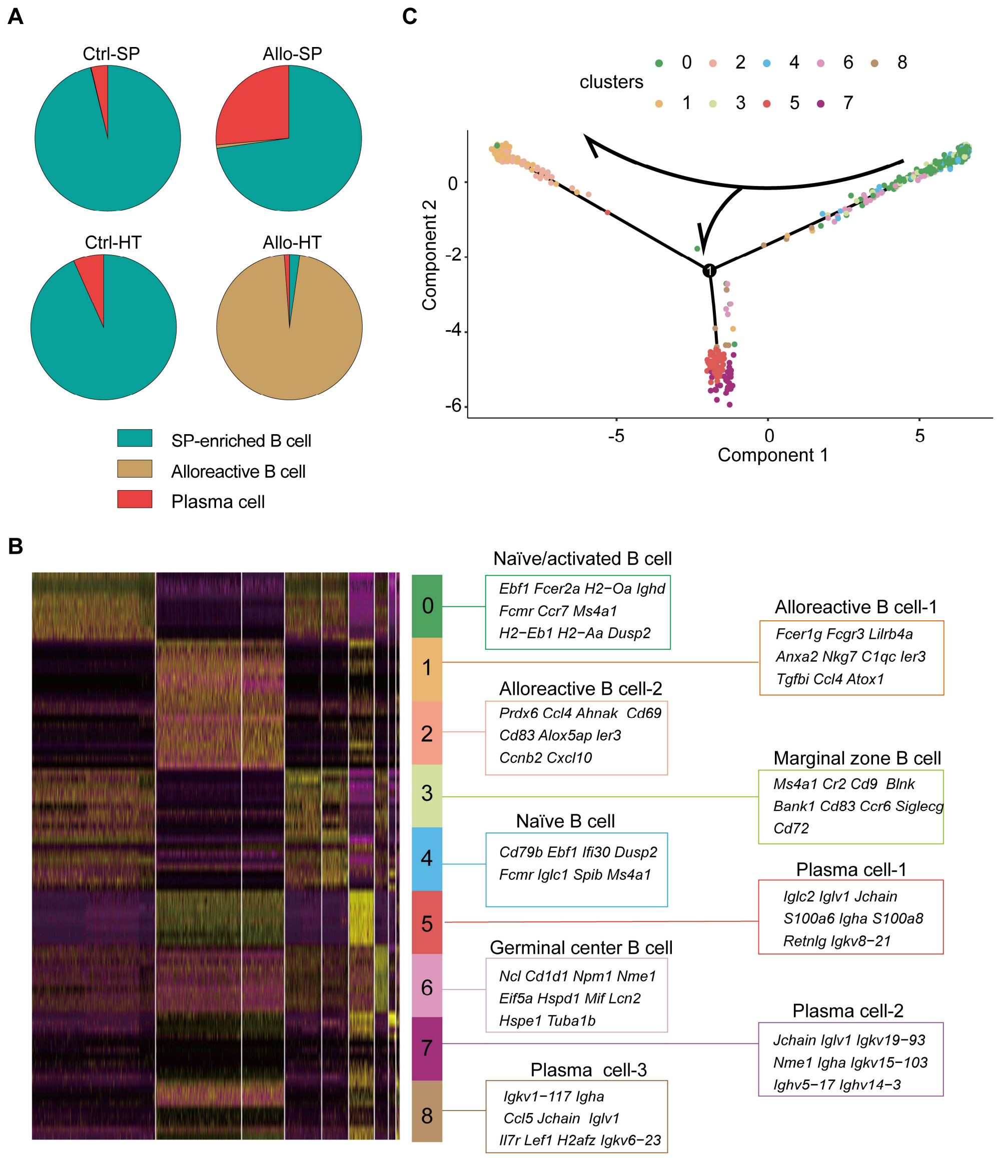

Figure S4. (Related to Figure 4) Focused analysis of B cells

(A) Pie charts demonstrating the proportion of three main B cell types in different groups. (B) Heatmap demonstrating expression of top 10 DEGs across cell clusters (columns). Top10 DEGs are also showed on the right with putative biological identity. (C) Monocle pseudotime inference traces a path of nine color-coded subclusters.

Figure S5

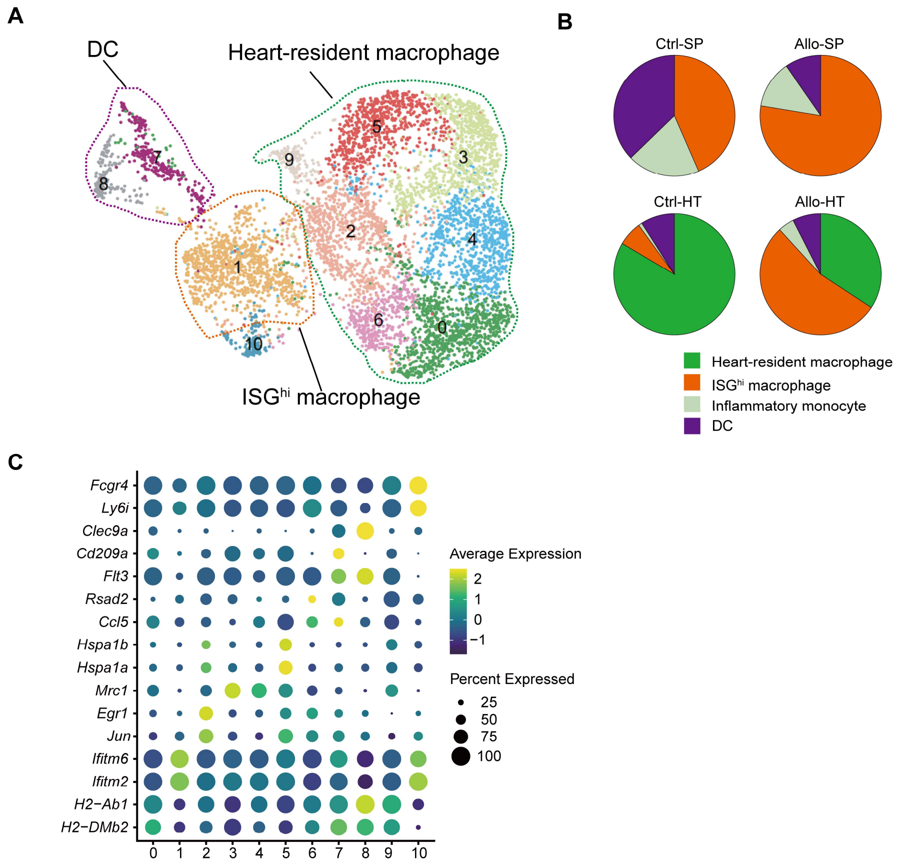

Figure S5. (Related to Figure 5) Focused analysis of Myeloid cells

(A) UMAP plots showing eleven color-coded cell subclusters of myeloid cells. (B) Pie charts demonstrating the proportion of four main cell types in different groups. (C) Dot plot showing expression levels (color-scaled, column-wise Z scores) of selected genes (columns)) and percentage of expressing cells (dot size) among eleven cell clusters (rows).

Figure S6

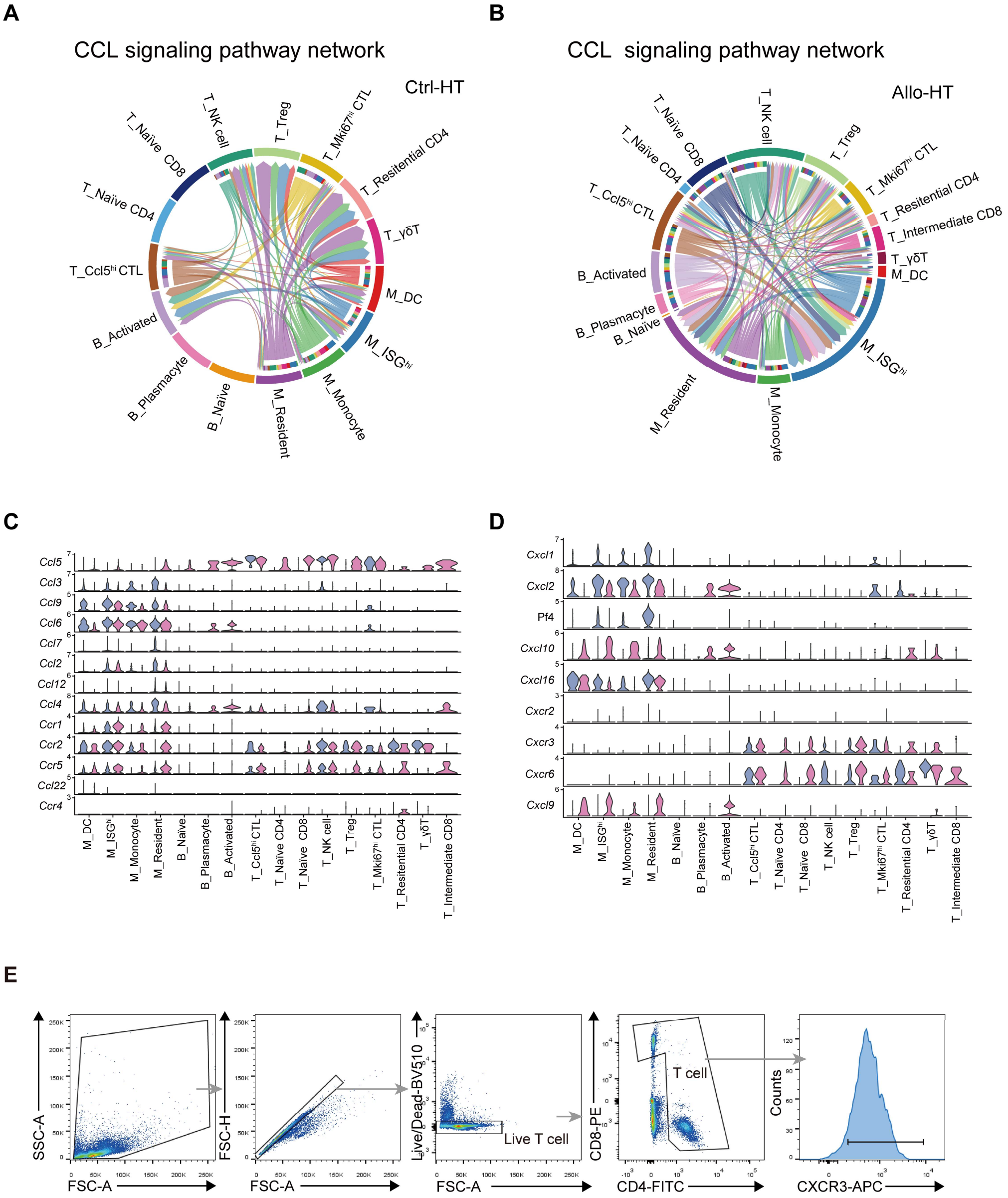

Figure S6. (Related to Figure 6) Local intercellular communication analysis

(A) CCL signaling pathway network in Ctrl-HT. (B) CCL signaling pathway network in Allo-HT. (C) Expression distribution of CCL pathway signaling genes at Ctrl-HT (blue) and Allo-HT (red). (D) Expression distribution of CXCR pathway signaling genes at Ctrl-HT (blue) and Allo-HT (red). (E) Representative Gating strategy of $CXCR3^+$ T cell.

Figure S7

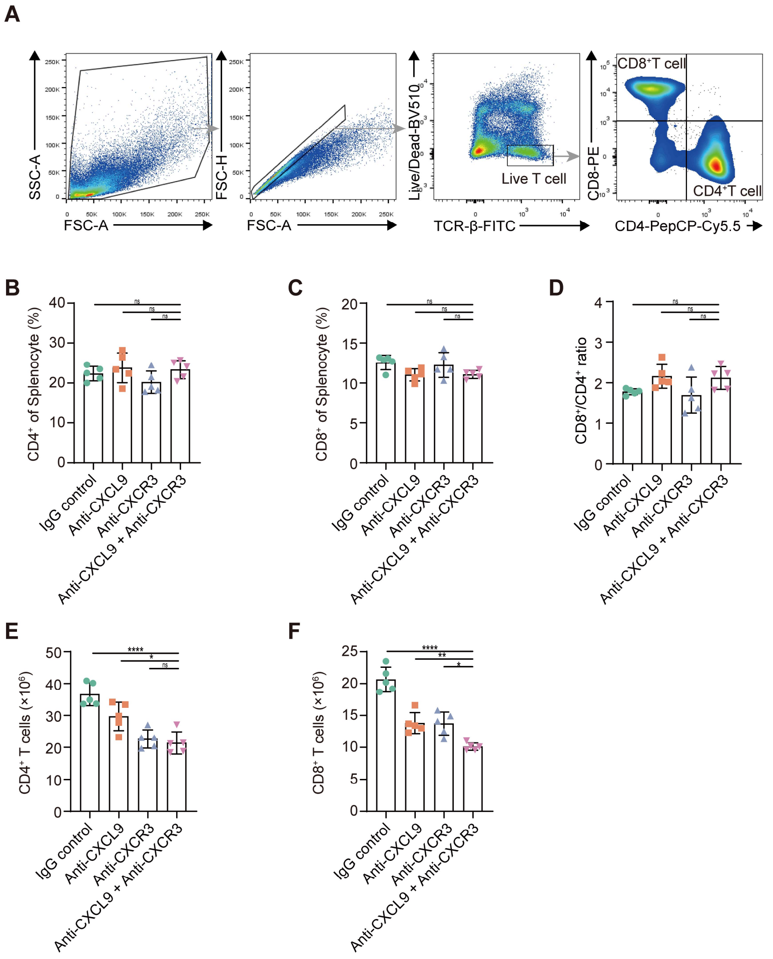

Figure S7. (Related to Figure 7) The impact of targeting CXCL9/CXCR3 axis to CD4 and CD8 cells.

(A) Representative Gating strategy of live CD4^{+} and CD8^{+} T cell. Samples are splenocytes which received at day 6 post-transplant. (B) Percentage of CD4^{+} of splenocyte at day 6post-transplant (n = 5). (C) Percentage of CD8^{+} of splenocyte at day 6 post-transplant (n = 5). (D) Bar Plots of CD8^{+}/CD4^{+} T cells ratio at day 6 post-transplant (n = 5). (E) The number of CD4^{+} T cells at day 6 post-transplant (n = 5). (F) The number of CD8^{+} T cells at day 6 post-transplant (n = 5). All data are representative of three independent experiments at least. One way ANOVA was used for comparisons among multiple groups. Data represented as mean ± SD. ns, not statistically significant. * $p < 0.05$, ** $p < 0.01$, *** $p < 0.001$, **** $p < 0.0001$

Figure S8

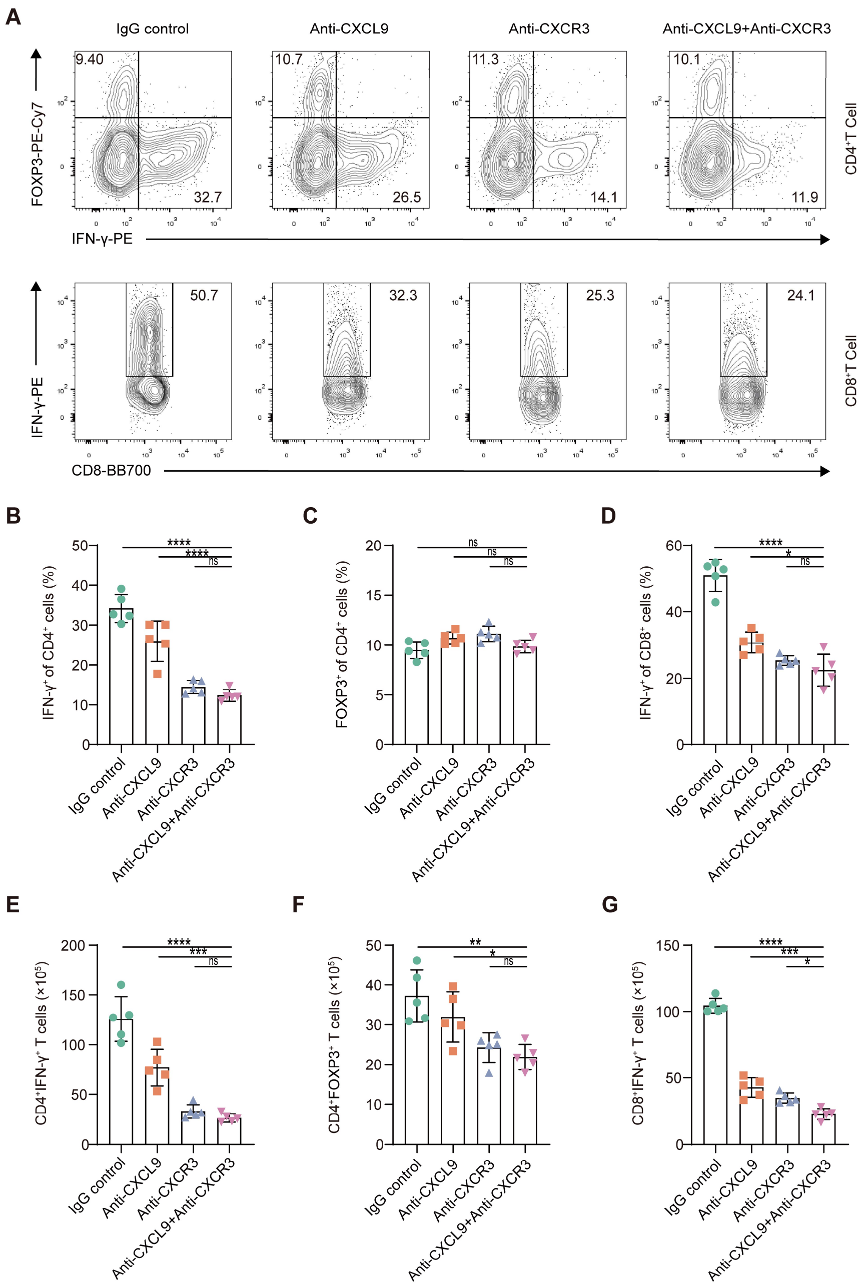

Figure S8. Effects of blocking the CXCR3 pathway on spleen T cell subsets in the murine heart transplantation model.

(A) Representative FCM plots show the percentage of FOXP3$^+$ or IFN-γ$^+$ cell in CD4 T cell and IFN-γ$^+$ cell in CD8 T cell at day 6 post-transplant. (B) Bar plots show the percentage of IFN-γ$^+$ cells in CD4 T cells at day 6 post-transplant (n = 5). (C) Bar plots show the percentage of Treg cells (FOXP3$^+$) in CD4 T cells (n = 5). (D) Bar plots show

the percentage of IFN-γ^+ cells in CD8 T cells (n = 5). (E) Bar plots show the number of $CD4^+IFN\text{-}\gamma^+$ T cells (n = 5). (F) Bar plots show the number of Treg cells (n = 5). (G) Bar plots show the number of $CD8^+IFN\text{-}\gamma^+$ T cells (n = 5). All data are representative of three independent experiments at least. One way ANOVA was used for comparisons among multiple groups. Data represented as mean ± SD. ns, not statistically significant. * $p < 0.05$, **$p < 0.01$, *** $p < 0.001$, **** $p < 0.0001$.

Table S1

Table S1 Key resources table

Reagent or resources	Source	Identifier
**Antibodies**		
Anti-CD8 (clone 53-6.7)	Biolegend (USA)	Cat# 100707
Anti-CXCR3 (clone S18001A)	Biolegend (USA)	Cat# 155905
Anti-CD4 (clone RM4-4)	Biolegend (USA)	Cat# 116011
Anti-CD44 (clone IM7)	Biolegend (USA)	Cat# 103019
Anti-TCR (clone H57-597)	Biolegend (USA)	Cat# 109205
Anti-CD45 (clone 30F11)	Biolegend (USA)	Cat# 103112
Anti-IFN-γ (clone XMG1.2)	Biolegend (USA)	Cat# 505807
Anti-CD4 (clone RM4-5)	Biolegend (USA)	Cat# 100515
Anti- Foxp3 (clone FJK-16s)	eBioscience (USA)	Cat# 25-5773-82
Anti-CD8 (clone 53-6.7)	BD Biosciences (USA)	Cat# 566409
InVivoMAb anti-mouse CXCR3 (clone CXCR3-173)	BioXCell (USA)	Cat# BE0249
InVivoMAb anti-mouse CXCL9 (clone MIG-2F5.5)	BioXCell (USA)	Cat# BE0309
InVivoPlus polyclonal Armenian hamster IgG	BioXCell (USA)	Cat# BP0091
**Critical Commercial Assays**		
Zombie Aqua Fixable Viability Kit	BioLegend (USA)	Cat# 423102
Fixation/Permeabilization Solution Kit	BD Biosciences (USA)	Cat# 554715
**Chemicals and enzyme**		
Percoll	Solarbio (China)	Cat# P8370
Collagenase B	Roche (USA)	Cat# 11088815001
Phorbol 12-myristate 13-acetate	Abmole (China)	Cat# M4647
Ionomycin	Sigma–Aldrich (USA)	Cat# 407951
GolgiStop	BD Biosciences (USA)	Cat# 554724
**Package and Database**		
Cell Ranger (version 3.0.2)	10× Genomics (USA)	https://support.10xgenomics.com/
Seurat (version 4.0.1)	Satija Lab (USA)	https://satijalab.org/seurat/
ClusterProfiler (version 3.18.1)	YuLab-SMU (China)	https://guangchuangyu.github.io /software/clusterProfiler/
Molecular Signatures Database (version 7.4)	UC San Diego and Broad Institute (USA)	https://www.gsea-msigdb.org/gsea/msigdb/team.jsp

Monocle2 (version 2.18.0)	Cole-trapnell-lab (USA)	http://cole-trapnell-lab.github.io/monocle-release/
CellChat (version 1.0.0)	Suoqin Jin Lab (China)	http://www.cellchat.org/
